# Supplementary material for: Influences of hand action on the processing of symbolic numbers: A special role of pointing?
Source: PLoS One. 2022 Jun 10;17(6):e0269557. doi: 10.1371/journal.pone.0269557 (PMC9187111; doi:10.1371/journal.pone.0269557)
Supplement: Attachment — Submitted filename: Review.pdf [file pone.0269557.s001.pdf]

The manuscript presents a novel and interesting study investigating the impact of hand action on the processing of symbolic numbers. The authors found slower reaction times in the number comparison task after repetitive pointing. The distance effect, a classical signature of semantic number processing, was diminished after performing pointing and enhanced after performing grasping. The study elegantly implies a classical neural adaptation paradigm, thus temporally dissociating manual actions and number processing. A study was pre-registered. Moreover, Bayesian statistical analysis was used to draw conclusions about both positive and negative findings. Methodologically speaking, this is a powerful and innovative study.

I do not have major concerns regarding the manuscript. However, several terminological clarifications and formatting improvements would be beneficial for the manuscript's clarity, and several relevant sources could enrich the interpretation of the findings.

Minor concerns:

1. The authors draw a parallel between nature vs. nurture / grounded vs. embodied cognition (line 46). As I understand these terms, nature relates here to inherited properties of the cognitive system, i.e., those that are genetically/biologically predefined. In contrast, grounded representations (i.e., an association between gravity and number/space) are still learned in the course of individual cognitive development. I would either clarify this parallel or remove it from the text as confusing.

2. Throughout the text (e.g., lines 45 vs. 51), the term grounded is used in two meanings: one is like in line 45 (grounded cognition, i.e., constraints of the physical world reflected in cognitive processes); the other one is like in line 51 ("cultural habits… grounded on cognitive and neural constraints", i.e., having neuronal or cognitive correlates). I would recommend the authors either make an explicit distinction between the two meanings or replace the term with a different one in one type of context.

3. The authors use the term number processes (e.g., line 67), which sounds unclear to me. I am more familiar with number processing or numerical processing. I would stick to these more established terms if this is what is meant. In that case, the expressions like "number processes… are grounded on neurocognitive constraints" (lines 67-68) could be reformulated as "numerical processing is constrained by neuronal mechanisms", which, in my opinion, is more theoretically precise. See also "process of numerical magnitude and distance" (lines 310-311) for the same problem.

4. The authors review the literature on the SNARC effect (lines 80-94), but only the part that investigated the effect in hand responses. However, spatial-numerical associations were also demonstrated for other effectors (eye movements, Myachykov et al., 2016; foot responses, Schwarz and Müller, 2006; full-body movements, see for reviews Fischer and Shaki, 2014; Toomarian and Hubbard, 2018). Whether numbers are associated with hand or lateralized space, this distinction is known in the literature as the location vs. effector hypothesis. It is discussed, for example, in this publication: Gut, M., Binder, M., Finc, K., & Szeszkowski, W. (2021). Brain activity underlying response induced by SNARC-congruent and SNARC-incongruent stimuli. Acta Neurobiol Exp, 81. It is worth mentioning that there is more support in favor of the location hypothesis. I do not insist that the authors refer to this literature in their introduction, but they might want to consider it in the discussion section.

5. The authors discuss pointing (lines 80-94) and grasping (lines 95-107) in numerical tasks; however, one of the references in the former para is about grasping (reference 35).

6. My impression is that the authors unnecessary parallel pointing vs. grasping with MNL vs. ATOM accounts: these two new concepts (MNL and ATOM) are not used in the analysis or interpretation of results and only appear once again in the introduction. I would recommend removing them entirely and speaking instead about ordinal vs. cardinal aspects of number meaning, like in this work (also relevant for the current study): Wiemers, M., Bekkering, H., & Lindemann, O. (2017). Two attributes of number meaning. Experimental psychology.

   Otherwise, the authors might want to relate their findings to these two theories (MNL and ATOM) in the discussion section.

7. It is also possible that the two aspects (ordinal vs. cardinal) diverge at the inter-individual level, i.e., some participants rely more on one, while other participants on the other aspect: see Krause, F., Lindemann, O., Toni, I., and Bekkering, H. (2013). Different brains process numbers differently: structural bases of individual differences in spatial and nonspatial number representations. J. Cogn. Neurosci. 26, 768–776. doi: 10.1162/jocn_a_00518

8. Unfortunately, I was not able to understand how exactly the randomization was performed (lines 240-245). In particular, the authors write here: “Consecutive blocks presenting the same action, were highly improbable cases.” – Does this mean that participants could potentially perform the same action block (tapping, pointing, etc.) several times? If not, then what exactly method was used to randomize blocks (e.g., true randomization, Latin square, etc.)?

9. I found it inconvenient to shuttle back and forth between the main text and a long detailed description of Fig. 1. I would recommend shifting all relevant details about the procedure to the main text while keeping the figure caption short.

10. It is written in the capture for Fig. 1: “a minimum of 1000ms and in any case after manual encoding of the participant’s response by the experimenter.” Does this mean that the experimenter initiated each trial, e.g., by a button press? If yes, this should be stated in the manuscript explicitly.

11. Through the text, the authors report SEM, even for descriptive statistics of accuracy (lines 289-291) or RT (Table 1). I am more familiar with reporting SDs in these contexts. Is there a particular reason for reporting SEM instead?

12. The authors introduce the term transport component, an average of grasping and pointing conditions (line 344). But tapping also includes hand movement to the object. It probably requires a better term or a justification for why tapping is not a part of the transport component.

13. It remained unclear to me how the clusters (Fig. 3c and 3d) were defined. Was a cluster analysis applied, or was a particular numerical threshold introduced on one or both variables? Since some observations from different clusters overlap on each variable, I assume the former. If this is the case, the clustering method should be described in detail. These figures are pretty complicated and are not referred to in Discussion. Perhaps, they could even be omitted in the manuscript if they do not add substantial evidence in favor of any of the hypotheses.

14. The authors hypothesize that ordinality is acquired earlier than cardinality (lines 440-443). I would refer here to a model of early mathematical development by Krajewski, which supports

this idea. See Fig. 1 at: Krajewski, K., & Schneider, W. (2009). Early development of quantity to number-word linkage as a precursor of mathematical school achievement and mathematical difficulties: Findings from a four-year longitudinal study. Learning and instruction, 19(6), 513-526.

15. In Conclusion, the authors claim that the study supports bidirectionality of action-number links (line 476). However, bidirectionality implies that numbers also influence actions. The authors did not test this direction in their study. I would remove this term.

16. I would recommend the authors summarize once again the main findings of the study in one or two sentences in Conclusion, right after the first sentence (line 477), and before discussing methodological advances and suggestions for future research.

17. I personally found it confusing to see a citation at the end of the conclusion, especially the one that includes “see also”. It makes an impression of an incomplete text. I would omit the last citations entirely, or if it is crucial for the authors to keep them, rephrase the sentence so that the references appear at its beginning.
